# Proposal of a form for the collection of videolaryngostroboscopy basic findings

**DOI:** 10.1007/s00405-018-4991-7

**Published:** 2018-05-22

**Authors:** Andrea Ricci-Maccarini, Giuseppe Bergamini, Rolando Fustos

**Affiliations:** 1 U.O. ORL, Ospedale “M. Bufalini”, Cesena, Italy; 2 P.C.M., Modena, Italy; 3 University of Rome “Cattolica”, Section “Claudiana”, Bolzano, Italy

**Keywords:** Laryngostroboscopy, Laryngostroboscopic parameters, Assessment of dysphonia, Diagnosis of laryngeal diseases

## Abstract

Videolaryngostroboscopy is a useful investigation required for a correct diagnosis of laryngeal diseases and voice disorders. We present a form for the collection of basic laryngostroboscopic findings, which provides for the evaluation of the classical six parameters codified by Hirano (symmetry and periodicity of glottic vibration, glottic closure, profile of vocal fold edge, amplitude of vocal fold vibration, mucosal wave) and six other parameters which we have included in the form for an essential and complete laryngostroboscopic evaluation (supraglottic framework behaviour, seat of phonatory vibration, vocal fold morphology and motility, level of the vocal fold, stops of vocal fold mucosa vibration). This form was created in 2002 during the elaboration of the protocol for the assessment of dysphonia of the Italian Society of Phoniatrics and Logopedics, which follows the guidelines of the European Laryngological Society published in 2001. We used this form for 15 years in our daily laryngological practice with great satisfaction. We propose a more detailed version of this form, which provides for drawings which show the various videolaryngostroboscopic findings, helping the laryngologist in the collection of videolaryngostroboscopic examination basic findings.

Videolaryngostroboscopy is one of the most widely performed examination in laryngological and in phoniatric fields. It associates the advantages of stroboscopic observation with the video recording of images and voice.

The stroboscopic effect is based on the particular functioning of the human eye, whereby an image remains imprinted in the retina for two-tenths of a second (Talbot’s law); if five images are given for subsequent glottic vibration cycles, they are “assembled” as if they were a single moving image. The selection of images to be “assembled” is made by light flashes synchronized with the glottic vibration frequency picked up by a microphone. The technological evolution of the various components (stroboscopic light sources, endoscopic investigation apparatus, computerized video recording instruments) has made it possible to increase the diagnostic sensitivity and fields of application of this examination. As Rosen [1] states, despite the limitation represented by the subjectivity of the interpretation of video images which reduces their applicability in the field of research, videolaryngostroboscopy is currently the most important clinical tool for diagnostic evaluation and subsequent therapeutic planning of patients with voice disorders. In fact the various pathological situations responsible for dysphonia almost always cause voice disorder through an alteration of glottic vibration. Obviously, this diagnostic tool requires adequate experience on the part of the examiner to capture the large amount of information that it can provide. Videolaryngostroboscopy has some limitations, in particular it is only possible with regular voice signal, time duration is needed and also a regular vibratory pattern. Videolaryngostroboscopy has become a useful examination to confirm the diagnostic suspicion of congenital and acquired vocal fold lesions; in these cases, when the voice signal is too irregular to capture a fundamental frequency for stroboscopy, or if the vibratory pattern is too irregular, high speed videolaryngoscopy is one of the main indications. The alteration of the mucosal wave is due to the adherence of the vocal fold epithelium to the vocal ligament, caused by the lack of the lax tissue which is normally contained in the superficial layer of the lamina propria, causing a stop of the mucosal wave [2], as it happens in the vocal fold scars and in laryngeal pre-neoplastic lesions [3–6].

The laryngostroboscopic examination provides the “slow-motion” mode, which allows the display of the vibration cycle in slow motion with variation of the speed of the displayed glottic vibration by means of a pedal and the “stand-still” mode, in which the displayed glottic vibration is fixed in the closing, semi-opening and opening phase.

The recording of the videolaryngostroboscopic examination is currently carried out in digital mode, with storing of the video recording in a host computer, so that it can be reviewed and compared with previous examinations.

The standard examination is usually performed with a rigid 70° or 90° optics; the new flexible endoscopes with distal chip camera allow to perform an examination with the same quality as rigid optics, but with greater comfort for the patient, as well as they allow a more physiological evaluation, since it does not involve the extrusion of the tongue.

Videolaryngostroboscopy is currently also performed in the operating room during thyroplasty or fiberendoscopic phonosurgery, with the possibility of immediate control of the results of phonosurgery on glottic vibration.

The main parameters of interpretation of the videolaryngostroboscopic examination are still those codified by Hirano and Cornut [2, 7]; in 2002 Bergamini, Ricci-Maccarini and Fustos during the development of the Protocol for Assessment of Dysphonia of the Italian Society of Phoniatrics and Logopedics (SIFEL Protocol), which follows the guidelines of the European Laryngological Society published in 2001 [8], elaborated a form which contains also other evaluation parameters, which complete the collection of essential findings obtained from the videolaryngostroboscopic examination, improving this important diagnostic tool. This form provides for the evaluation of 12 parameters, which include the 6 classical parameters codified by Hirano [7] and 6 other parameters which complete the clinical-instrumental investigation (Fig. 1). This form was subsequently extended in 2008 in the occasion of the Official lecture at 32° National Congress of the Association of the Italian Hospital Otorhinolaryngologysts, but 15 years of use in phoniatric clinical practice have resulted in our continuing to use the version contained in the SIFEL Protocol, since this is simple, quick to perform and it contains all the essential evaluations for a complete videolaryngostroboscopic examination.

**Fig. 1:**
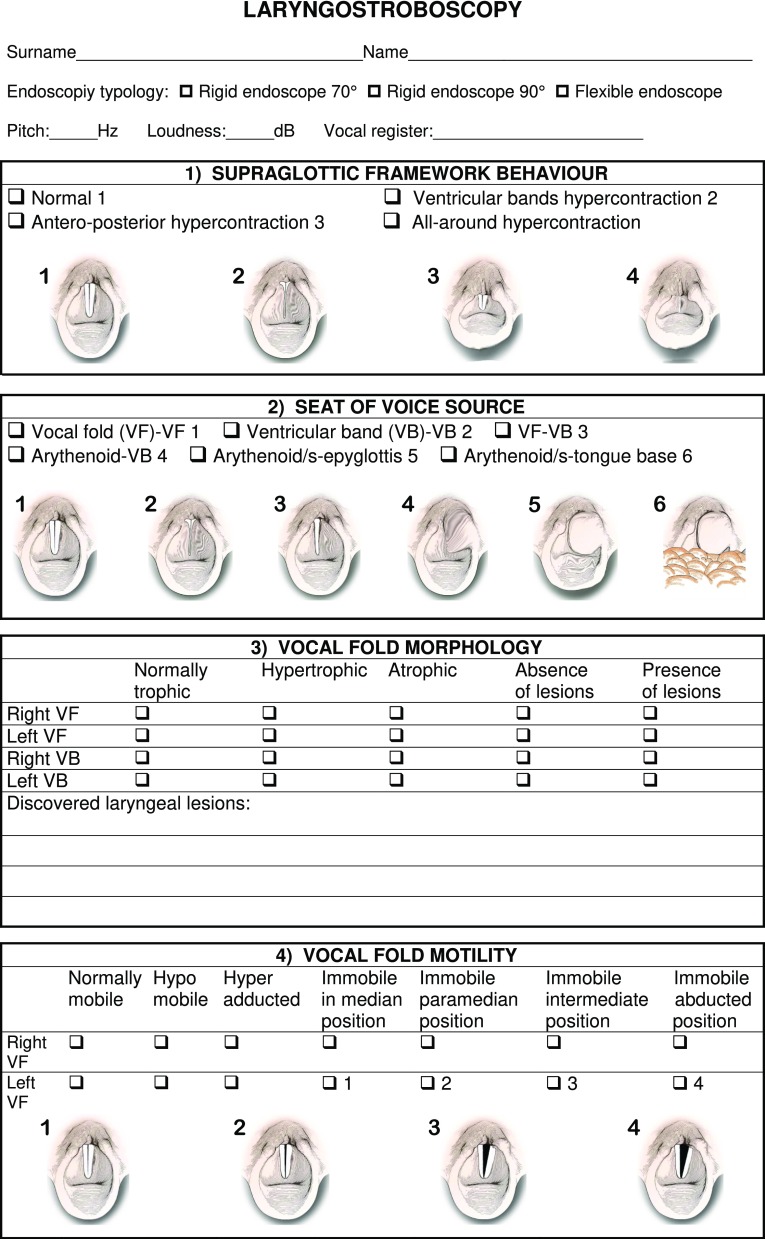

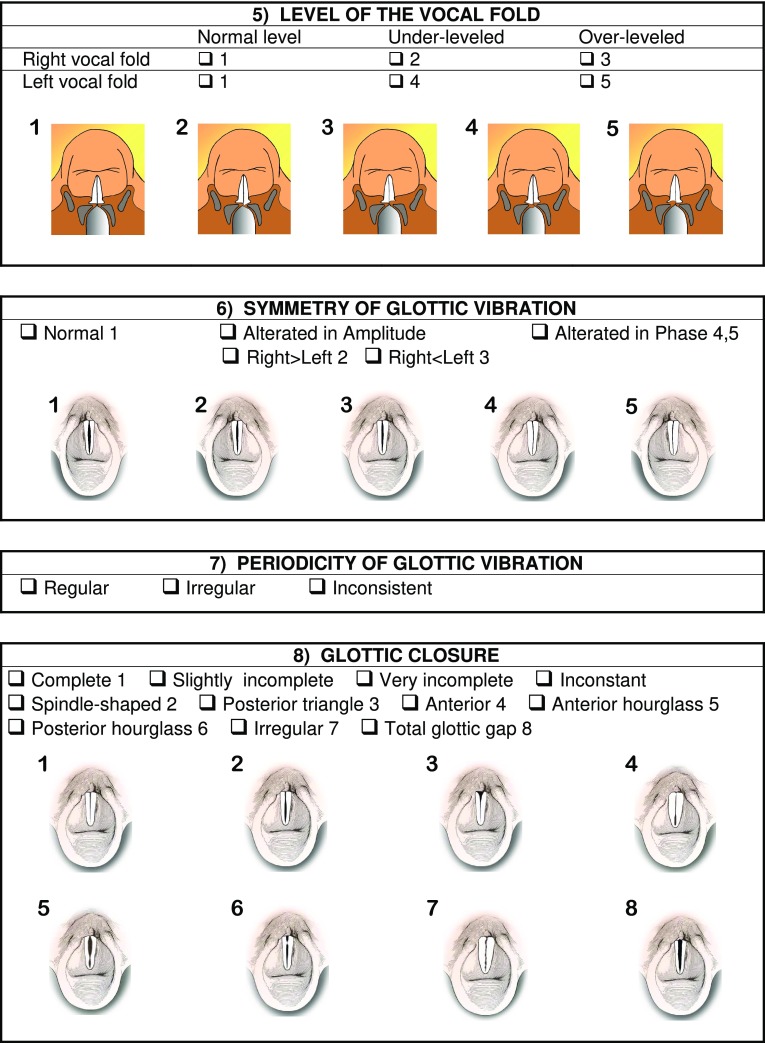

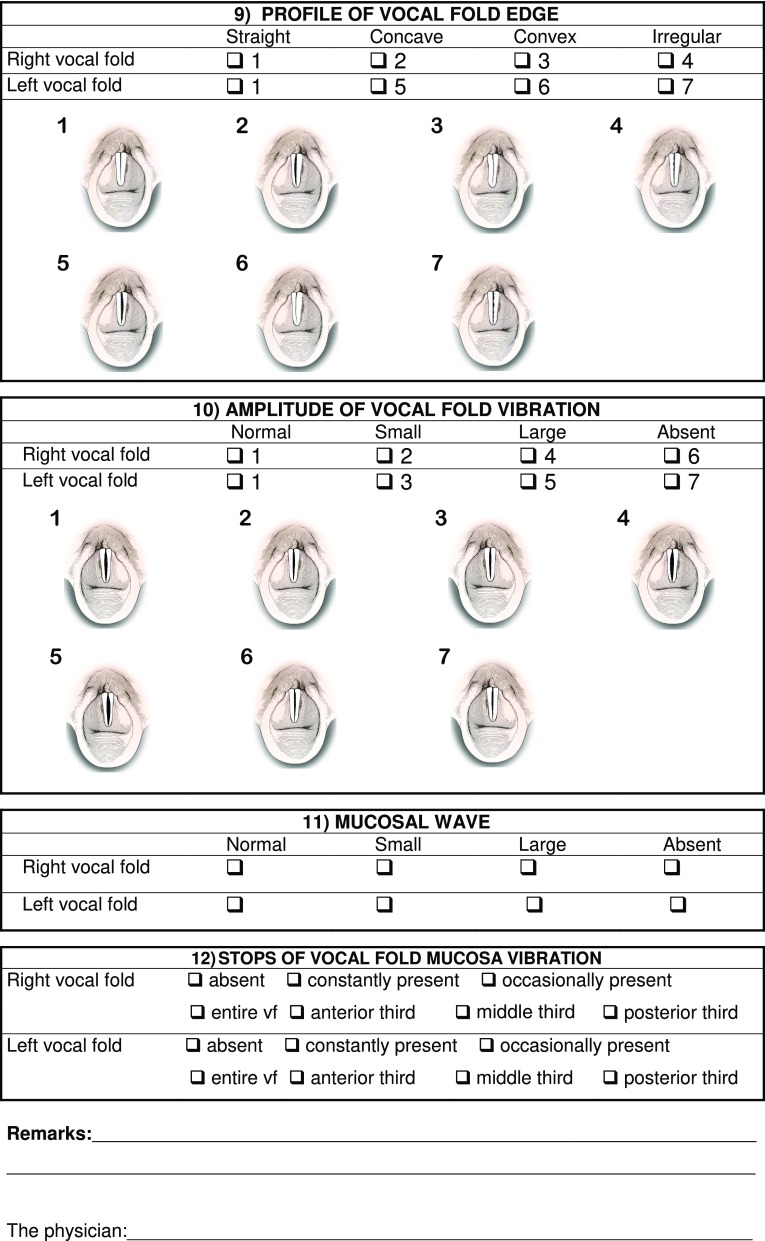
Form with the collection of the 12 laryngostroboscopic parameters

In the present work we have added, for each parameter contained in the form, the schematization of the relative videolaryngostroboscopic image, to provide the examiner with a complete working tool without any doubts of interpretation.

In addition to the findings relating to the 12 laryngostroboscopic parameters contained in the form, some lines are also provided for the annotation of remarks regarding other particular findings which may be useful for performing a more complete videolaryngostroboscopic examination.

Also noted are the type of endoscope used, rigid or flexible, the mean fundamental frequency in Hz of the vowel produced during the examination, its loudness in dB and the vocal register (modal or falsetto).

We now illustrate the 12 parameters contained in the form, shown in Fig. 1, which can be digitally filed so as to obtain a database for clinical comparisons between examinations performed on the same patient, for example before and after a treatment and statistical comparisons between examinations performed on different patients.

1. Supraglottic framework behaviour

This parameter, introduced during the elaboration of the form included in the SIFEL Protocol, provides for: the presence of normal behaviour of supraglottic structures during phonation; or the presence of hypercontraction of ventricular bands with their possible vibratory contact; or an anteroposterior supraglottic hypercontraction, with possible vibratory contact between the arytenoids and the epiglottis foot; or an all-round hypercontraction of supraglottic structures.

This parameter allows detecting the presence of a compensatory supraglottal hypercontraction of a glottal insufficiency of an organic or functional type in a framework of hyperkinetic dysphonia.

2. Seat of voice source

This parameter also has recently been introduced in the SIFEL protocol. It is particularly useful in the evaluation of cordectomy or partial laryngectomy outcomes [5]. The vibratory contact can normally be between vocal fold and vocal fold; or between the ventricular bands, possibly at the same time as the glottic vibration (bitonal voice); or between vocal fold and ventricular band; or between arytenoid and ventricular band; or between the arytenoids and between arytenoid/s and epiglottis; or between arytenoid/s and tongue base, in outcomes of supracricoid or supratracheal partial laryngectomy, when the vocal folds, ventricular bands and epiglottis have been removed [5].

3. Vocal fold morphology

This parameter is useful for annotating the presence of normal morphology of the vocal folds and ventricular bands, with absence of lesions; or the presence of hypertrophy or atrophy of the vocal folds and/or ventricular bands; or the presence of laryngeal lesions, with some lines for describing the type and location of the discovered lesions.

4. Vocal fold motility

This is an indispensable parameter in the diagnosis of laryngeal paralysis. The vocal fold can be normally mobile, hypomobile, hyperadducted during phonation (as compensation for a paralysis of the contralateral vocal fold), immobile; in case of immobility, the vocal fold can be immobile in median, paramedian, intermediate or abducted position. This parameter allows a correct evaluation of the results obtained by phonosurgery after the medialization of a vocal fold which is immobile in intermediate or abducted position, with glottic insufficiency during phonation.

5. Level of the vocal fold

It is another important parameter for the evaluation of vocal fold paralysis. The immobile vocal fold can be at a normal level compared to the contralateral normomobile vocal fold, or it can be under-levelled (in most cases) or over-levelled. This parameter must be evaluated by means of a flexible endoscope, possibly rotating the videocamera by 180° (or inverting the image in case of distal chip-camera flexible endoscope), to obtain an image “from behind” which permits better appreciating the level difference between the two vocal folds.

6. Symmetry of glottic vibration

It is a classic parameter codified by Hirano [7]. It can be normal when the opening phase of the two vocal folds during glottic vibration has the same amplitude; or it can be altered in amplitude when a vocal fold has a less wide opening phase than the other vocal fold (e.g. due to the presence of an intracordal cyst); or there may be an asymmetry in phase, when during glottic vibration one vocal fold is in opening phase while the other is in closing phase (e.g., in some cases of muscle tension dysphonia). For a correct evaluation of this parameter, it is useful to perform the laryngostroboscopic examination both in “slow-motion” and “stand-still” mode; the latter allows fixing the various phases of the glottic vibratory cycle, highlighting any phase and/or amplitude asymmetry.

7. Periodicity of glottic vibration

A classic parameter codified by Hirano [7]. It can be regular, irregular or inconsistent. The laryngostroboscopic examination in stand-still mode shows, in case of irregular glottic vibration, an unclear image of the various phases of the vibratory cycle, since it is not repeated in the same way cycle after cycle. The evaluation of inconsistent glottic vibration is a limit of laryngostroboscopy; in these cases high-speed laryngeal endoscopy [9] is preferable as this allows visualizing all the cycles of glottic and/or supraglottic vibration, regular, irregular or inconsistent, with a slower but real image (not “reconstructed” as in the laryngostroboscopy) of 2 s of phonatory vibration, with time delay and without sound.

8. Glottic closure

It is a classic parameter codified by Hirano [7]. It is one of the fundamental parameters of videolaryngostroboscopy, essential in the diagnosis of glottic insufficiency.

Glottic closure can be complete, incomplete or inconstant (sometimes complete and sometimes incomplete). The incomplete glottic closure may be slightly incomplete or very incomplete, with glottic gap morphology that may be: spindle-shaped, posterior triangle, anterior, anterior hourglass, posterior hourglass, irregular (due to the presence of neoformations affecting the vocal fold edge), total (which affects the entire length of the glottis).

9. Profile of vocal fold edge

Parameter codified by Hirano [7]. It can be straight, concave, convex or irregular (due to the presence of neoformations).

10. Amplitude of vocal fold vibration

A classic parameter codified by Hirano [7]. It can be normal, small (as in benign intracordal lesions), large (as in denervated and flaccid vocal folds) or absent (as in neoplastic infiltration of the vocalis muscle) [3, 4, 6]. The evaluation of vocal fold vibration amplitude must be kept distinct from that of the mucosal wave.

11. Mucosal wave

It is a classic parameter codified by Hirano [7], it is one of the fundamental parameters of laryngostroboscopy; it evaluates the progression of the wave generated by the flowing of the “cover” (vocal fold epithelium) on the “body” (vocal ligament and vocalis muscle) due to the lax tissue contained in the superficial layer of the lamina propria, driven by subglottic pressure, myoelastic and aerodynamic forces [7]. The mucosal wave may be absent due to the presence of adherence between epithelium and vocal ligament with a vocal fold vibration always present albeit reduced, when the vocalis muscle is normal, as in precancerous vocal fold lesions and in early glottic cancer [3, 4, 6] or in benign vocal fold lesions such as the deep vergeture and the iatrogenic vocal fold scars [2]; or mucosal wave can be small or large.

12. Stops of vocal fold mucosa vibration

Parameter introduced during the development of the SIFEL Protocol. It defines, more specifically, the areas of adherence of the vocal fold mucosa where the mucosal wave stops. Vibration stops may occur constantly or occasionally and they may affect the anterior third, the middle third or the posterior third of the vocal fold, or they may affect the entire vocal fold.

At the end of the videolaryngostroboscopic examination, together with the specialist report, the four most significant images are printed, in which the vocal folds are in breathing position, in glottic closure, in half-opening and in opening phase.

We recommend a more widespread use of the videolaryngostroboscopic examination in laryngological clinical practice and the use of this form to collect the examination findings, to obtain a correct laryngological diagnosis and evaluation parameters which can be compared with those obtained from the patient at different times, especially before and after medical treatment, voice therapy or phonosurgery. This form is particularly useful for the training of young laryngologysts, while for the expert laryngologysts it could be too long to apply; a shortened version of the form will be elaborated for this aim in the future.
